# Bioactive Compounds of Cooked Tomato Sauce Modulate Oxidative Stress and Arachidonic Acid Cascade Induced by Oxidized LDL in Macrophage Cultures

**DOI:** 10.3390/nu11081880

**Published:** 2019-08-13

**Authors:** Carolina E. Storniolo, Ignasi Sacanella, María T. Mitjavila, Rosa M. Lamuela-Raventos, Juan J. Moreno

**Affiliations:** 1 Department of Nutrition, Food Sciences and Gastronomy, School of Pharmacy and Food Sciences, University of Barcelona, 08921 Barcelona, Spain; 2 Institute of Nutrition and Food Safety (INSA-UB), University of Barcelona, 08921 Barcelona, Spain; 3 Department of Immunology, Physiology and Cell Biology, School of Biology, University of Barcelona, 08028 Barcelona, Spain; 4 CIBER Fisiopatología de la Obesidad y la Nutrición (CIBEROBN), Instituto de Salud Carlos III, 28029 Madrid, Spain

**Keywords:** tomato, olive oil, superoxide, nitric oxide, prostaglandin E_2_, leukotriene B_4_

## Abstract

*Sofrito* is a mix of tomato, onion, garlic, and olive oil, which contains phenolic compounds and carotenoids. Consumption of tomato-based sofrito has been related to a lower risk of cardiovascular events, but the mechanisms behind such beneficial effects remain unclear. This study aimed to analyze the effects of representative *sofrito* compounds such as naringenin, hydroxytyrosol, lycopene, and β-carotene on mechanisms involved in the pathogenesis of atherosclerosis. We demonstrated that both phenolic compounds and both carotenoids studied were able to inhibit low density lipoproteins (LDL) oxidation, as well as oxidative stress and eicosanoid production induced by oxidized LDL (oxLDL) in macrophage cultures. These effects were not the consequences of disturbing oxLDL uptake by macrophages. Finally, we observed an additive effect of these *sofrito* compounds, as well as the activity of a main naringenin metabolite, naringenin 7-*O*-β-d-glucuronide on LDL oxidation and oxidative stress.

## 1. Introduction

Epidemiologic and prospective studies have provided strong evidence that a diet rich in a variety of fruits and vegetables, such as the Mediterranean diet, results in a lower risk of developing cardiovascular diseases (CVD) [1,2,3]. In this way, tomato and tomato-based products have been associated with decreased levels of cardiovascular risk factors [4,5,6]. However, the bioactive compounds and the mechanisms behind such beneficial effects remain unclear.

*Sofrito* is not a unique recipe but usually contents a mix of ingredients such as tomato, onion, garlic and olive oil, which contains many bioactive phenolic compounds and carotenoids [7]. Naringenin and chalcone naringenin, a naringenin precursor, are accepted as the main flavonoids in tomatoes and tomato-based sauces [8,9], whereas lycopene and β-carotene are the more representative carotenoids [7] and hydroxytyrosol appears in *sofrito* and tomato sauces prepared with olive oil [7]. Interestingly, *sofrito* has higher levels of bioactive compounds than tomato. Thus, 120 g of sofrito added to different dishes daily, leads to a total phenolic intake of 15–25 mg and 5–10 mg of carotenoids. Recently, it was reported that some of these compounds could modulate oxidative stress and eicosanoid production [10], involved in chronic processes with an important inflammatory component, such as CVD.

The protective beneficial effects of tomato sauces have been mainly attributed to their capacity to prevent atherogenesis by their antioxidant properties [11,12]. However, few studies have demonstrated anti-atherogenic effects for narigenin [13] or lycopene [14]. Thus, the action of *sofrito* bioactive compounds on the main mechanisms involved in CVD remains to be investigated. Low-density lipoproteins (LDL) oxidation is an early and critical event in atherogenesis [15,16]. However, high levels of oxidized LDL (oxLDL) correlate with the severity of CVD [17], and oxLDL uptake by macrophages promotes the recruitment of leukocytes and accumulation of lipid-laden macrophages named foam cells [18], which are the most predominant cell type in the fatty streaks [19].

Considering the above mentioned observations, we aimed to study the effect of compounds found in *sofrito* on oxidation of LDL, as well as on oxidative stress and eicosanoid production by macrophages incubated in the presence of oxLDL, important events implicated in the pathogenesis of atherosclerosis.

## 2. Materials and Methods

### 2.1. Materials and Chemicals

Naringenin, lycopene, β-carotene, hydroxytyrosol, 1,1′-dioctadecyl-3,3,3′,3′ tetra-methylindocarbocyanine perchlorate (Dil), p-nitro blue tetrazolium (NBT), phorbol myristate-acetate (PMA), superoxide dismutase (SOD), catalase, and fucoidan were obtained from Sigma Chemical Co. (St. Louis, MO, USA). Naringenin 7-*O*-β-d-glucuronide was obtained from Cayman Chem. Co. (Ann Arbor, MI, USA). Cell culture medium, foetal bovine serum and supplements were obtained from GE Healthcare Hyclone (Logan, UT, USA). All other reagents were of analytical grade. Assayed compounds were dissolved in dimethylsulfoxide (DMSO) and the final concentration of DMSO in cell cultures was lower than 0.1%.

### 2.2. Culture of Human Monocyte/Macrophage 

Human monocyte cell line THP-1 was obtained from American Type Culture Collection (ATCC, Manassas, VA, USA). The cells were maintained at a maximal density of 1 × 10^6^ cells/mL in RPMI 1640 medium containing 10% fetal bovine serum and 1% penicillin/streptomycin solution at 37 °C in a 5% CO_2_ incubation. To induce macrophage differentiation, THP-1 cell (1 × 10^5^ cells/well) were incubated in multi-well plates in the presence of 100 nM PMA for 48 h to complete the morphological and biochemical differentiation to macrophages [20].

### 2.3. Isolation of LDL and LDL Oxidation Assay

LDL (density 1.03–1.053) were prepared by sequential ultracentrifugation from pooled, citrated human plasma from six healthy normolipidemic volunteers (personnel of the laboratory), according to the method of Chung et al. [21]. Finally, LDL were dialysed, filtered through a 0.45 µM filter and stored at 4 °C. The protein concentration was determined by the Lowry method modified by Peterson [22]. 

LDL were diluted with PBS to a final concentration of 50 µg protein/mL and incubated at 37 °C with freshly prepared CuSO_4_·5H_2_O (2.5 µM) in the presence of the tested compounds. Conjugated dienes (CD) were monitored spectrophotometrically (234 nm) every 5 min as described by Esterbauer et al. [23] and thiobarbituric acid reactive substances (TBARs) measured fluorimetrically (excitation at 515 nm and emission at 553 nm) according to the method described by Yagi [24].

### 2.4. Assay for Binding and/or Uptake of oxLDL by Macrophages

In order to elucidate whether the effects observed were a consequence of disturbing oxLDL uptake by macrophages, oxLDL were labelled using the fluorescent probe Dil according to the methodology described by Innerarity et al. [25]. Thus, oxLDL (1 mg protein/mL) were incubated overnight with Dil (30 µg/mL) at 37 °C and, finally, Dil-labelled oxLDL were isolated by ultracentrifugation. The medium was removed and macrophages were incubated in the presence of studied compounds and finally Dil-labelled oxLDL (50 µg protein/mL) or unlabelled oxLDL (50 µg protein/mL) were incubated for 3 h. After being washed three times with PBS, cells were lysed with 0.1 M NaOH, and the solution was neutralised with 0.1 M HCl. Fluorescence intensity was measured with a spectrofluorometer (excitation at 524 nm and emission at 567 nm) [26].

### 2.5. Determination of Reactive Oxygen Species (ROS) and Nitric Oxide (NO) Production

The medium was removed from macrophage cultures, which were incubated with the testing compounds for 30 min and then stimulated with oxLDL (50 µg protein/mL) for 30 min. Superoxide anion (O_2_^−^) generation was determined in aliquots of culture supernatant by measuring the superoxide dismutase-inhibitable reduction of NBT [27]. Nitrite (NO_2_^−^) accumulation in the cell-free medium, an indicator of NO production, was measured using the Griess reagent as we described previously [27].

### 2.6. Measurement of Prostaglandin E_2_ (PGE_2_) and Leukotriene B_4_ (LTB_4_)

The medium of macrophage cultures was removed and testing compounds were incubated for 30 min, and then macrophages were stimulated with oxLDL (50 µg protein/mL) for 30 min. An aliquot of culture medium (0.25 mL) was acidified with 1 mL of 1% formic acid. PGE_2_ or LTB_4_ was extracted in ethyl acetate (5 mL) and, after discarding the aqueous phase, the organic phase was evaporated under a stream of nitrogen. PGE_2_ and LTB_4_ levels were determined using monoclonal enzyme immunoassay kits (Cayman Chemical Co., Ann Arbor, MI, USA) following the manufacturer’s protocol.

### 2.7. Statistical Analysis

Results are expressed as mean ± SEM. Differences between control and treated samples were tested using either Student’s *t*-test or one-way analysis of variance, followed by the least significant differences test when appropriate differences were considered statistically significant at *p* < 0.05.

## 3. Results

### 3.1. Sofrito Compounds Modulate LDL Oxidation Induced by Cu^2+^

To mimic in vivo conditions, pooled plasma was preincubated with the compounds under study at 37 °C for 1 h. LDL were subsequently isolated and its resistance to oxidation induced by Cu_2_SO_4_ tested. CD and TBARs production were reduced by the presence of bioactive compounds, and hydroxytyrosol and naringenin being more effective than lycopene or β-carotene (Table 1). Our results show an additive effect of these compounds at 1 µM that was able to delay LDL oxidation.

Bioactive compound concentrations was chosen considering the plasma and tissue levels reached after *sofrito* ingestion [8].

### 3.2. Phenolic Compounds and Carotenoids of Sofrito Reduce Oxidative Stress and Eicosanoid Synthesis Induced by oxLDL

OxLDL (50 µg protein/mL) induced O_2_^−^ and NO_2_^−^ formation with maximum levels reached 30 min after macrophages stimulation (Table 2). We observed no cell toxicity using this range of concentration of oxLDL and the exogenous addition of superoxide dismutase (SOD) or catalase inhibited oxidative stress induced by oxLDL in these experimental conditions (data not shown). Furthermore, we observed that oxLDL were able to induce cyclooxygenase and 5-lipoxygenase pathways reflected in the enhancement of PGE_2_ and LTB_4_ synthesis in macrophage cultures (Table 2). 

**Figure 1:**
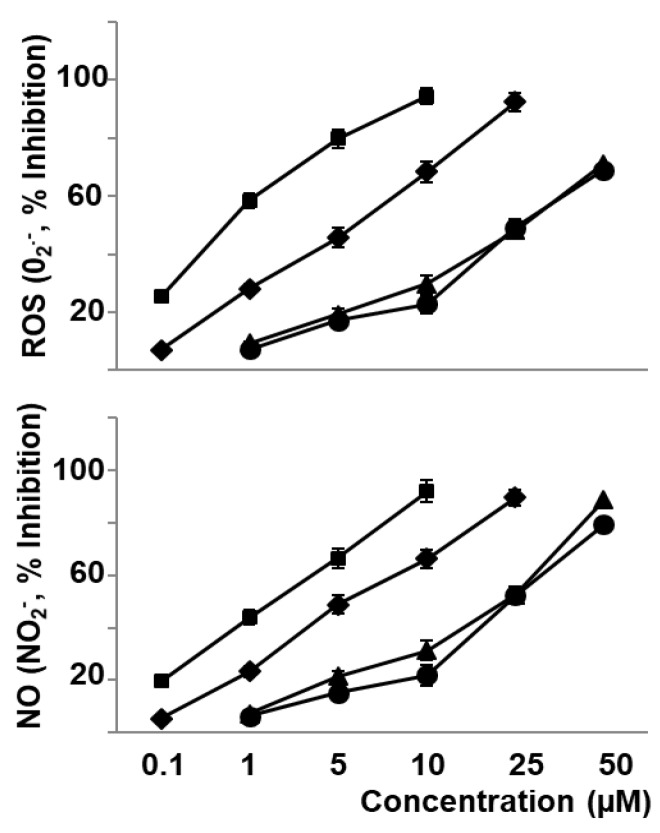
Effect of bioactive compounds of *sofrito* on O_2_^−^ and NO_2_^−^ production stimulated by oxLDL. Macrophages were incubated with naringenin (♦), hydroxytyrosol (■), lycopene (▲), or β-carotene (●) for 30 min, and then stimulated with oxLDL (50 µg protein/mL) for 30 min. Finally, O_2_^−^ and NO_2_^−^ were measured. Data are the means ± SEM of three experiments performed in triplicate.

**Figure 2:**
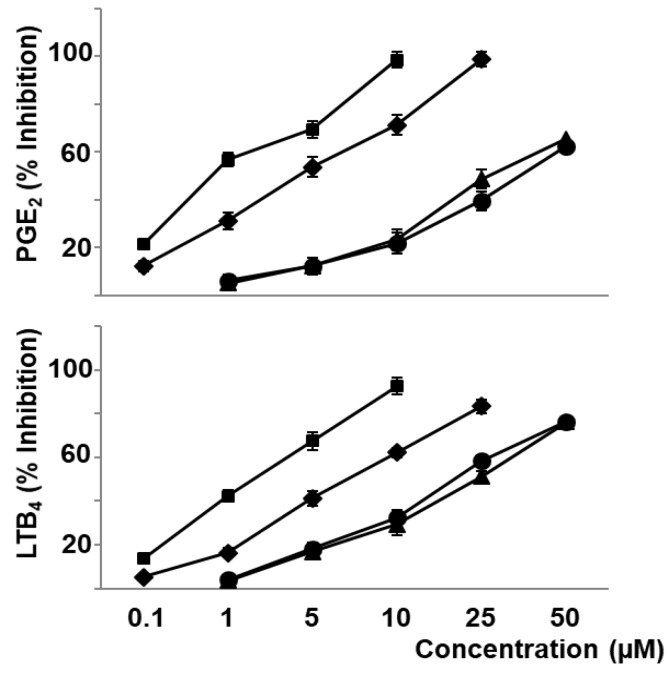
Effect of bioactive compounds of *sofrito* on prostaglandin E_2_ and leukotriene B_4_ production stimulated by oxLDL. Macrophages were incubated with naringenin (♦), hydroxytyrosol (■), lycopene (▲), or β-carotene (●) for 30 min, and then stimulated with oxLDL (50 µg protein/mL) for 30 min. Finally, PGE_2_ or LTB_4_ were measured. Data are the means ± SEM of three experiments performed in triplicate.

The present work shows that naringenin (0.1–25 µM), hydroxytyrosol (0.1–10 µM), lycopene (1–50 µM), and β-carotene (1–50 µM) decreased in a concentration-dependent manner the production of O_2_^−^ and NO_2_^−^ produced by oxLDL stimulated macrophages, as indicators of ROS and NO production, respectively (Figure 1). In a similar form, these compounds decreased the oxLDL induced biosynthesis of eicosanoids representative of cyclooxygenase and 5-lipoxygenase pathways such as PGE_2_ and LTB_4_, respectively (Figure 2). 

**Figure 3:**
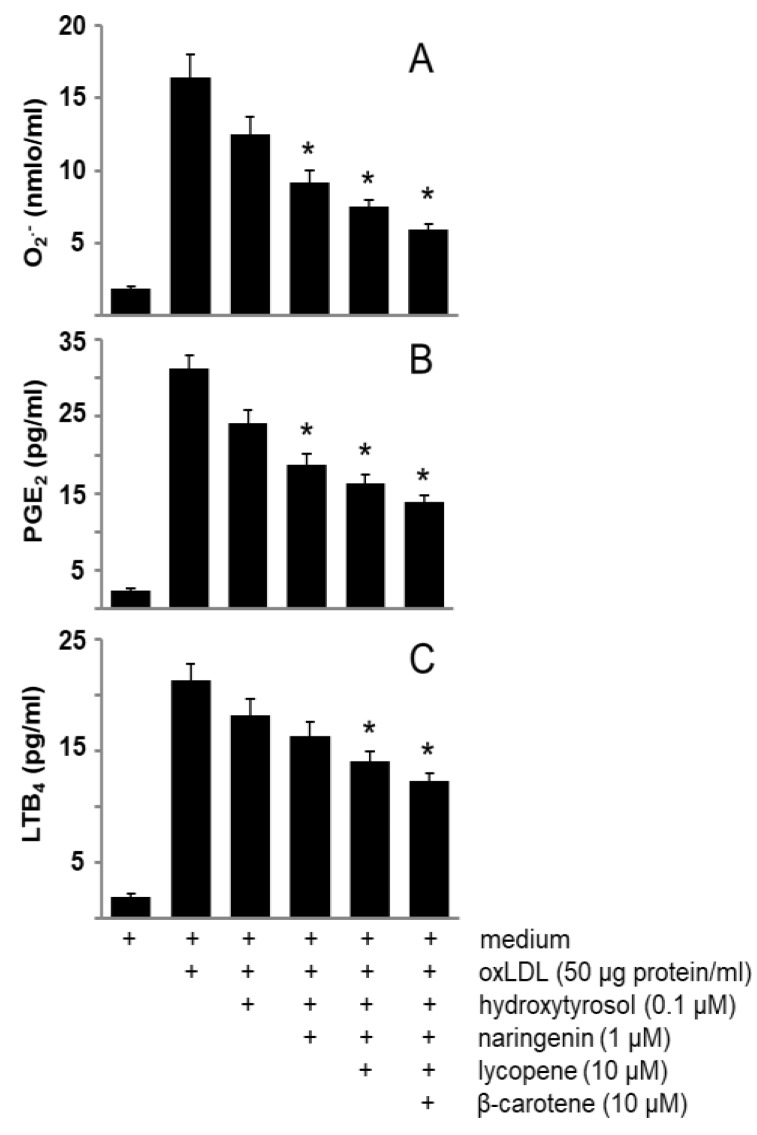
Effects of bioactive compounds of *sofrito* on O_2_^−^, PGE_2_ and LTB_4_ production stimulated by oxLDL. Macrophages were incubated with hydroxytyrosol (0.1 µM), naringenin (1 µM), lycopene (10 µM) or β-carotene (10 µM) for 30 min, and then stimulated with oxLDL (50 µg protein/mL) for 30 min. Finally, O_2_^−^ (**A**), PGE_2_ (**B**) or LTB_4_ (**C**) were measured. Data are the means ± SEM of three experiments performed in triplicate. * Significantly different (*p* < 0.05) with respect to oxLDL-stimulated macrophages.

Interestingly, we did not observe any effect of these compounds on non-stimulated cells (data not shown), but we observed an additive effect of these compounds on O_2_^−^, PGE_2_, and LTB_4_ formation induced by oxLDL (Figure 3).

### 3.3. Phenolic Compounds and Carotenoids of Sofrito Did Not Affect the Binding and Uptake of oxLDL by Macrophages

We also examined the effects of these compounds found in *sofrito* on the binding and uptake of oxLDL using Dil-labelled oxLDL. As shown in Table 3, incubation of macrophages with Dil-labelled oxLDL (50 µg protein/mL) resulted in a marked increase in fluorescence intensity, while autofluorescence of the sample prepared from cells stimulated with unlabelled oxLDL, was less than 10 units, findings that demonstrated an oxLDL uptake by macrophages. Under these conditions, naringenin (10 µM), hydroxytyrosol (10 µM), lycopene (50 µM), or β-carotene (50 µM) did not modify fluorescence intensity induced by Dil-labelled oxLDL, whereas fucoidan (10 µg/mL), a polyanionic polysaccharide, effective competitor for oxLDL in studies of receptor binding [28], prevented the increase in fluorescence intensity. Thus, the above effects induced by oxLDL on oxidative stress and eicosanoid production were not consequence of any disturbance of oxLDL binding to macrophages.

### 3.4. Naringenin 7-O-β-d-glucuronide Has Similar Activity on Oxidative Stress and Eicosanoid Biosynthesis to Naringenin

Naringenin, once absorbed, is conjugated with sulphates and glucuronides by the enterocytes and transported to the liver where it is also rapidly conjugated as other polyphenols [29]. Hence, it is important to evaluate if naringenin’s antioxidant activity and other biological activities are maintained after losing one or more hydroxyl groups of naringenin. Our results show that naringenin 7-*O*-β-d-glucuronide inhibits LDL oxidation induced by Cu^2+^ (Table 1) as well as oxidative stress and eicosanoid synthesis induced by oxLDL in a similar extent to naringenin (Table 2).

## 4. Discussion

Atherosclerosis is thought to involve the uptake of oxLDL by macrophages and vascular wall cells [30,31]. The generation of ROS and NO [32], as well as pro-inflammatory mediators such as eicosanoids [33]. Characteristic bioactive compounds of *sofrito* such as hydroxytyrosol, naringenin, lycopene, and β-carotene have been demonstrated to be effective as free radical scavengers [34,35,36]. Thus, the first question addressed by the present study was whether these bioactive compounds present in *sofrito* were able to modulate LDL oxidation. Our findings demonstrated that both phenolic compounds (hydroxytyrosol and naringenin) and both carotenoids (lycopene and β-carotene) effectively increased resistance to Cu^2+^-mediated LDL oxidation, being phenolic compounds more effective than carotenoids. Interestingly, the simultaneous incubation of these compounds at concentrations that can be reached in plasma after *sofrito* ingestion [37] induced a significant slow/inhibition of LDL oxidation. These findings suggest an important additive and beneficial effect of bioactive *sofrito* compounds on this event with a pivotal role in early steps of atherogenesis, as was previously reported using lycopene in combination with other bioactive compounds [38].

The present work also shows that exposure to low, non-toxic levels of oxLDL, leads to the production of ROS, NO, and eicosanoids by human macrophages obtained by differentiation of THP-1 monocytes, in agreement with previous results obtained using murine macrophages RAW 264.7 [39]. Interestingly, these effects were observed when we examined an oxLDL concentration capable of transforming macrophages into foam cells [40], a pivotal element in the development of atheroma plaque.

Although several authors reported that olive oil or tomato phenolic compounds and carotenoids modulate LDL oxidation or eicosanoid synthesis using different experimental models, the present study is the first to quantitatively compare the effect of compounds found in *sofrito* on oxidative stress and eicosanoid synthesis induced by oxLDL in macrophage cultures. The redox state of the cell may act as a molecular switch regulating different genes and enzymes. Previously, we demonstrated that changes of redox state of macrophages stimulated arachidonic acid cascade and consequently the biosynthesis of eicosanoids [41,42,43]. In this way, Lupo et al. [44] demonstrated that oxLDL induced Ca^2+^-dependent as well as Ca^2+^-independent phospholipase A_2_, enzymes involved in arachidonic acid release and consequently in eicosanoid synthesis. Furthermore, Muroya et al. [45] described that oxLDL induced NFκB signalling, a redox-sensitive transcription factor involved in the regulation of cyclooxygenase pathway in macrophages, a pivotal element in PGE_2_ synthesis. Considered all together, we hypothesized that oxidative stress and eicosanoid synthesis induced by oxLDL can be modulated by the *sofrito* compound tested. This hypothesis is supported by the fact that naringenin, hydroxytyrosol, lycopene, and β-carotene were able to inhibit O_2_^−^, NO_2_^−^, PGE_2_ and LTB_4_ production induced by oxLDL. Obviously, sofrito is not a unique recipe throughout the Mediterranean countries and consequently the bioactive compound profile can be more diverse. However, the above mentioned compounds can be considered representative of this dish. The more interesting findings of the present study are the additive effects observed when co-incubating macrophages with the phenols and carotenoids tested on the modulation of oxidative stress and eicosanoid synthesis induced by oxLDL. Thus, the simultaneous effects of *sofrito* bioactive compounds could result in an additional atheroprotective effect. *Sofrito* preparation involves cooking often at a high temperature that can induce oxidation. However, tomato cooking/processing maintains phenolic content and antioxidant activity [46]. Interestingly, such effects take place at bioactive compound concentrations within the range expected from nutritional intake of *sofrito* in the context of a Mediterranean type diet [37]. Furthermore, we must consider that these bioactive compounds have been also described to modulate inflammatory elements involved in atherogenesis such as cytokine release and adhesion molecules expression [47]. Other phenolic compounds or carotenoids can also potentially contribute to the above beneficial effects of *sofrito* on cardiovascular risk factors, especially when *sofrito* was cooked and enriched with olive oil [47]. Here, our results suggest that the healthy effects of *sofrito* enriched with olive oil can be, at least in part, a consequence of the presence of extra virgin olive oil rich phenolic compounds such as hydroxytyrosol.

Considering that oxLDL uptake and foam cell formation take place via scavenger receptors [48] and that Miles et al. [49] described that olive oil, a component of *sofrito,* decreased macrophage uptake of oxLDL though the down regulation of scavenger receptors, we attempted to explore whether *sofrito* compounds influenced oxLDL interaction with macrophages in our experimental conditions. Our findings demonstrated that the above effects of naringenin, hydroxytyrosol, lycopene, and β-carotene were not due to interference with oxLDL binding to macrophage receptors. However, we can not exclude that other bioactive compounds of *sofrito* could have this action. Thus, squalene present in olive oil inhibits oxLDL uptake by macrophages, by reducing CD36 expression whereas tyrosol and hydroxytyrosol did not have this effect [50], findings in agreement with our observations. 

The maximum concentrations of naringenin and its metabolites generally appear in 30–60 min after ingestion, and it found conjugated naringenin rather than its free form predominates in the circulation [37]. Consequently, considerable controversy exists as to whether naringenin, or other polyphenols, is an active molecule in vivo as its plasma concentration is in the nanomolar range compared with the micromolar range of its metabolites. Previously, we demonstrated that resveratrol metabolites are antioxidants and have similar biological effects to resveratrol [51]. Our findings demonstrated that naringenin and naringenin 7-*O*-β-d-glucuronide, to a similar extent, reduce the oxidative stress and eicosanoid synthesis induced by oxLDL and consequently both molecules may act with additive effects on mechanisms with a central role in atherogenesis.

## 5. Conclusions

Recently, Tresserra-Rimbau and co-workers analysed the most relevant current knowledge of bioactive polyphenols from culinary world to the clinical setting [52]. Here, our findings propose a new molecular mechanism by which bioactive compounds of *sofrito*, a characteristic ingredient of the Mediterranean diet, may prevent the biosynthesis of pro-inflammatory and pro-atherogenic mediators by oxLDL-stimulated macrophages. Most relevantly, we demonstrated an important additive effect of several *sofrito* compounds at physiological concentration, which can explain the beneficial effects of cooked tomato sauce, as it was recently reported by Vilahur et al. [53] on coronary endothelial function of dyslipidemic animals. Finally, we also observed that naringenin metabolites, which reach higher plasma concentrations than naringenin after oral ingestion, could be active to the same extent as the parent compound. Obviously, additional experimental assays and clinical trials are necessary to confirm the beneficial effects of *sofrito* inclusion in the diet.

## Figures and Tables

**Table 1:** Effect of *sofrito* bioactive compounds of on Cu^2+^ mediated oxidation of low-density lipoproteins LDL, which was monitored by conjugated dienes (CD) and thiobarbituric acid reactive substances (TBARs) formation. T_lag_ was the difference in lag phase (min) between Cu^2+^ and Cu^2+^ plus tested compounds. ΔT_lag_ (%) = sample T_lag_ − control T_lag_/control T_lag_ (× 100). MDA is expressed as nmol/mg of LDL protein. Superoxide dismutase (SOD) (30 µM) and catalase (50 µM) were used as positive control. Results are expressed as means ± SEM of two experiments performed in triplicate. * Significantly different from Cu^2+^ without treatments.

	CD		TBARs
	T_lag_ (min)	ΔT_lag_ (%)	MDA
LDL			3.2 ± 0.2
LDL + Cu^2+^	58 ± 6		27.3 ± 1.3
LDL + Cu^2+^ + Naringenin (1 µM)	97 ± 7 *	67.2	19.5 ± 0.7 *
LDL + Cu^2+^ + Naringenin (10 µM)	198 ± 13 *	241.4	12.1 ± 0.4 *
LDL + Cu^2+^ + Naringenin Gluc. (10 μM)	204 ± 12 *	251.7	13.6 ± 0.3 *
LDL + Cu^2+^ + Hydroxytyrosol (1 μM)	225 ± 11 *	287.9	14.8 ± 0.3 *
LDL + Cu^2+^ + Hydroxytyrosol (10 μM)	>500 *		8.3 ± 0.2 *
LDL + Cu^2+^ + Lycopene (10 μM)	73 ± 6	25.8	22.5 ± 0.4
LDL + Cu^2+^ + Lycopene (50 μM)	121 ± 8 *	108.6	15.7 ± 0.3 *
LDL + Cu^2+^ + β Carotene (10 μM)	72 ± 5	24.1	23.8 ± 0.4
LDL + Cu^2+^ + β Carotene (50 μM)	119 ± 7 *	105.2	17.9 ± 0.3 *
LDL + Cu^2+^ + all compounds (1 μM)	386 ± 8 *	565.5	10.6 ± 0.2 *
LDL + Cu^2+^ + SOD + Catalase	>500 *		5.7 ± 0.2 *

**Table 2:** Effects of naringenin and naringenin 7-*O*-β-d-glucuronide on reactive oxygen species (ROS), NO), PGE_2_ and leukotriene B_4_ (LTB_4_) production by macrophages in the presence of oxidized LDLs (oxLDLs). ROS are expressed as O_2_^−^ (nmol/mL), NO was indirectly determined as NO_2_^−^ (nmol/mL), and PGE_2_ and LTB_4_ concentration in cell culture supernatant were expressed as pg/mL. Naringenin and naringenin 7-*O*-β-d-glucuronide (10 µM) were incubated for 30 min and then stimulated with oxLDL for 30 min a 37 °C. Data are the means ± SEM. of three experiments performed in triplicate. * *p* < 0.05 versus control, ^≠^ *p* < 0.05 versus non-treated cells.

	ROS	NO	PGE_2_	LTB_4_
Control	2.0 ± 0.1	1.3 ± 0.1	2.5 ± 0.5	1.8 ± 0.4
oxLDL	15.4 ± 1.6 *	32.1 ± 2.7 *	26.8 ± 2.6 *	21.2 ± 1.8 *
oxLDL + Naringenin	6.3 ± 0.8 ^≠^	12.4 ± 1.3 ^≠^	11.6 ± 2.1 ^≠^	13.5 ± 1.4 ^≠^
oxLDL + Naringenin 7-*O*-β-gluc.	7.6 ± 1.2 ^≠^	13.6 ± 1.5 ^≠^	13.2 ± 2.5 ^≠^	15.1 ± 1.3 ^≠^

**Table 3:** Effect of *sofrito* compounds on binding and/or uptake of Dil-labelled oxLDL. Macrophages were incubated with the compounds for 1 h, and then incubated with Dil-oxLDL (50 µg protein/mL) for 3 h. Then, the cells were washed and lysated and the fluorescence intensity in the lysates were measured. Data are the means ± SEM of three experiments performed in duplicate. * Significant different from non-treated cells incubated with Dil-oxLDL.

	Fluorescence Intensity (Arbitrary Units)
oxLDL	21.2 ± 1.5
Dil-oxLDL	532.2 ± 21.3
Dil-oxLDL + Naringenin (10 µM)	476.7 ± 16.1
Dil-oxLDL + Hydroxytyrosol (10 µM)	453.4 ± 17.3
Dil-oxLDL + Lycopene (50 µM)	503.5 ± 13.8
Dil-oxLDL + β-Carotene (50 µM)	517.1 ± 12.8
Dil-oxLDL + Fucoidan (10 µg/mL)	102.6 ± 3.7 *
